# Regulation of Sulfotransferase and UDP-Glucuronosyltransferase Gene Expression by the PPARs

**DOI:** 10.1155/2009/728941

**Published:** 2009-08-10

**Authors:** Melissa Runge-Morris, Thomas A. Kocarek

**Affiliations:** Institute of Environmental Health Sciences, Wayne State University, Detroit, MI 48201, USA

## Abstract

During phase II metabolism, a substrate is rendered more hydrophilic through the covalent attachment of an endogenous molecule. The cytosolic sulfotransferase (SULT) and UDP-glucuronosyltransferase (UGT) families of enzymes account for the majority of phase II metabolism in humans and animals. In general, phase II metabolism is considered to be a detoxication process, as sulfate and glucuronide conjugates are more amenable to excretion and elimination than are the parent substrates. However, certain products of phase II metabolism (e.g., unstable sulfate conjugates) are genotoxic. Members of the nuclear receptor superfamily are particularly important regulators of SULT and UGT gene transcription. In metabolically active tissues, increasing evidence supports a major role for lipid-sensing transcription factors, such as peroxisome proliferator-activated receptors (PPARs), in the regulation of rodent and human SULT and UGT gene expression. This review summarizes current information regarding the regulation of these two major classes of phase II metabolizing enzyme by PPARs.

## 1. Introduction

**Figure 1:**
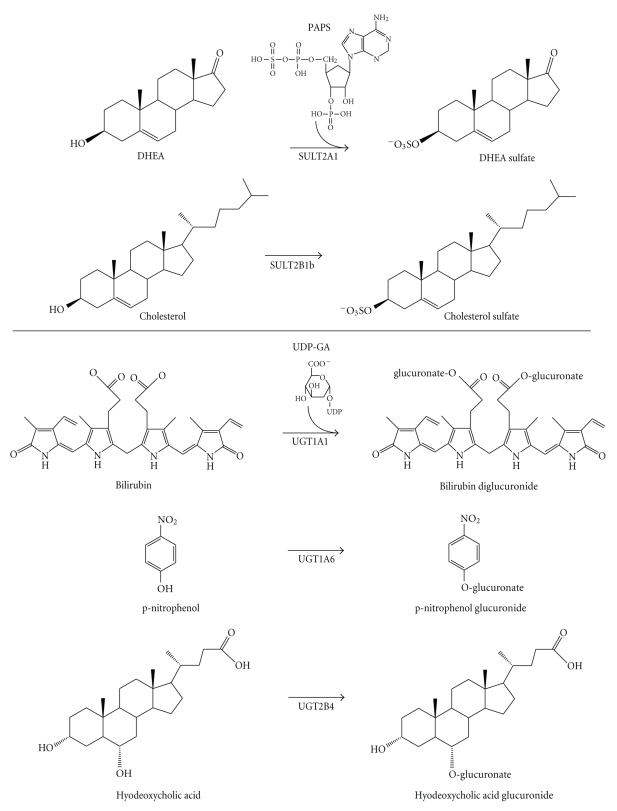
*Example reactions catalyzed by PPAR-regulated SULT and UGT enzymes. * The upper panel shows the SULT2A1- and SULT2B1b-catalyzed 3-sulfonation of the prototype substrates, dehydroepiandrosterone and cholesterol, respectively. Human SULT2A1 is transcriptionally regulated by PPAR*α* in human hepatocytes, while SULT2B1b is regulated by PPAR*α*, PPAR*δ* and PPAR*γ* in keratinocytes. The lower panel shows the glucuronidation of bilirubin, *p*-nitrophenol and hyodeoxycholic acid, which are prototype substrates for UGT1A1, UGT1A6, and UGT2B4, respectively. UGT1A1, UGT1A3, UGT1A4, UGT1A6, UGT1A9, and UGT2B4 have all been identified as PPAR target genes. PAPS, 3′-phosphoadenosine-5′-phosphosulfate; UDP-GA, uridine-5′-diphospho-*α*-D-glucuronic acid.

Phase II, or conjugative, metabolism is defined as the covalent attachment of an endogenous molecule to a functional group on a substrate molecule. Although a substrate containing a suitable functional group can directly undergo phase II metabolism, conjugation often occurs subsequent to a phase I reaction (e.g., catalyzed by a cytochrome P450), during which the functional group is added to the substrate. The conjugating moiety is most often a sulfonate or glucuronate group, although other conjugating moieties include glutathione, glycine, acetate, and the methyl group. Phase II metabolism usually increases the hydrophilicity of the substrate molecule, which facilitates transport and elimination of the product. Phase II sulfonation and glucuronidation reactions are catalyzed by the cytosolic sulfotransferase (SULT) and the UDP-glucuronosyltransferase (UGT) families of enzymes, respectively, (Figure 1). The SULT and UGT enzymes represent a highly responsive defense system against the mutagenicity of carcinogenic environmental chemicals and the toxicity of xenobiotics and endogenous metabolic intermediates. Members of the nuclear receptor superfamily are particularly important regulators of UGT and SULT gene transcription. In metabolically active tissues, increasing evidence supports a major role for lipid-sensing transcription factors, such as peroxisome proliferator-activated receptors (PPARs), in the regulation of rodent and human SULT and UGT gene expression. This review summarizes current information regarding the regulation of these two major classes of phase II metabolizing enzyme by PPARs.

## 2. PPARs

**Figure 2:**
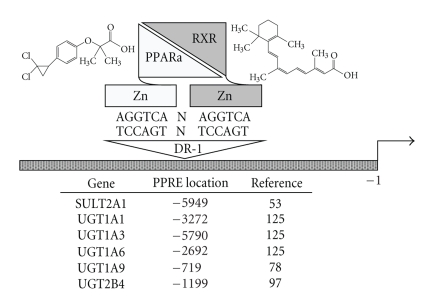
*Regulation of SULT and UGT gene transcription by* *PPARα*. The PPAR*α* and RXR nuclear receptors are each depicted as a zinc module-containing DNA-binding domain (rectangle) that is joined to a ligand-binding domain (LBD, triangle) through a hinge region. PPAR*α* and RXR bind as a heterodimer to a peroxisome proliferator response element (PPRE) in the regulatory region of a target gene. The consensus PPRE is a nuclear receptor hexamer motif (i.e., (A/G)G(G/T)TCA) in a DR-1 configuration (direct repeat with one intervening nucleotide, N). Binding of an agonist to the LBD of PPAR*α* (e.g., chemical structure for the potent PPAR*α* agonist, ciprofibrate, is shown) evokes a conformational change in the receptor that results in coactivator recruitment and increased target gene transcription. In contrast to its silent role in partnership with some nuclear receptors, RXR functions as an active partner with the PPARs, whereby binding of an agonist to the LBD of RXR (e.g., chemical structure for the prototype RXR ligand, 9-*cis*-retinoic acid, is shown) activates target gene transcription and enhances PPAR ligand-activated transcription. The locations of functional PPREs that have been identified in the 5′-flanking regions of the human *SULT2A1*, *UGT1A1*, *UGT1A3*, *UGT1A6*, *UGT1A9, * and *UGT2B4* genes are shown (positions are relative to the transcription start site).

The PPAR nuclear receptor network represents a central determinant of cellular energy balance. In heterodimeric partnership with the retinoid X receptor (RXR), PPAR forms a ligand-activated nuclear receptor transcription factor that is capable of integrating the expression of a wide spectrum of target genes involved in cellular lipid metabolism, energy homeostasis, and inflammation (Figure 2). The three known PPAR isoforms, PPAR*α*, PPAR*δ* (also called PPAR*β*) and PPAR*γ* are products of separate genes and are well conserved across species. PPAR*α* expression is the most prominent in the liver, kidney, and heart where it is engaged in the regulation of fatty acid oxidation [1]. By contrast, PPAR*γ* is most widely expressed in adipose tissues where it plays a role in adipocyte differentiation and the control of insulin sensitivity [1]. PPAR*δ* is ubiquitously expressed and has been implicated as a regulator of a range of physiological functions from the modulation of insulin resistance to embryo implantation during pregnancy [1]. Fibrates and thiazolidinediones are well-characterized ligand-activators of PPAR*α* and PPAR*γ*, respectively, [1]. Fatty acids represent a major source of cellular energy and are important physiological activators of PPAR*α* [1]. Relative to fatty acids, oxidized fatty acid intermediates are more short-lived species and thus are well poised to serve as both endogenous signaling intermediates and physiological PPAR*γ* agonists [2]. As a key integrator of cellular energy metabolism in a wide spectrum of tissues, the PPAR*·*RXR transcription factor network is being increasingly recognized for its potential as a therapeutic target and for its expanded role in gene regulation.

## 3. SULTs

### 3.1. The Role of SULTs in Metabolism

Sulfonation reactions are catalyzed by two distinct families of enzymes, the cytosolic SULTs and the membrane-bound sulfotransferases. Of these, only the SULTs participate in phase II drug metabolism; the membrane-bound sulfotransferases catalyze the sulfonation of proteins and complex carbohydrates [3]. The SULTs catalyze the transfer of a sulfonate moiety (SO_3_^−^) from the physiological donor 3′-phosphoadenosine-5′-phosphosulfate (PAPS) to a small molecule substrate containing a nucleophilic moiety such as a susceptible hydroxyl group [4] (Figure 1). The SULTs are widely expressed in hepatic and extrahepatic tissues [5] where they represent key components of the xenobiotic defense system. They also function prominently in physiological processes by metabolizing endogenous substrates, including estrogens [6], thyroid hormones [7], bile acids [8], and neurosteroids [9–12]. In xenobiotic metabolism, sulfate conjugation is recognized as a double-edged sword. As a rule, sulfate conjugates are more polar than the parent substrate and, hence, more amenable to excretion and elimination. However, the production of unstable sulfate conjugates can lead to the focused generation of genotoxic species and carcinogen activation [13, 14].

Like other classes of xenobiotic-metabolizing enzymes, the SULTs exist as a superfamily of related proteins with each enzyme exhibiting a characteristic expression pattern and substrate specificity profile. The cytosolic SULTs are categorized into two major groups, the arylsulfotransferases (SULT1 family) and the hydroxysteroid sulfotransferases (SULT2 family) [5, 15]. The SULT1 family is divided into five subfamilies, designated SULT1A, SULT1B, SULT1C, SULT1D, and SULT1E. As a brief generalization, SULT1A subfamily enzymes metabolize phenolic substrates and function in drug metabolism. For example, SULT1A1 readily catalyzes the sulfonation of simple phenols, such as 1-naphthol and *p*-nitrophenol [4], and detoxifies common phenolic pharmaceuticals, such as acetaminophen [16, 17] and troglitazone [4]. Consistent with its role in drug metabolism, SULT1A1 is abundantly expressed in liver, although it is also expressed in numerous extrahepatic tissues [5]. Rodent enzymes of the SULT1B subfamily catalyze the sulfonation of 3,5,3′-triiodothyronine [18], an important step in thyroid hormone metabolism, although SULT1A1 appears to perform this function in humans [19, 20]. SULT1C enzymes are best known for their abilities to bioactivate the heterocyclic amine procarcinogen, N-hydroxy-2-acetylaminofluorene, to its ultimate carcinogenic form [21]. SULT1D enzymes, described thus far chiefly in the canine, sulfonate phenols, amines, and eicosanoids [22]. SULT1E1 is the physiologic estrogen sulfotransferase, catalyzing the 3-sulfonation of estradiol at nanomolar concentrations [5]. SULT1E1 is expressed most highly in tissues that are actively engaged in estrogen metabolism, such as breast, uterus, and placenta [23–25].

The SULT2 family is divided into two subfamilies, SULT2A and SULT2B. In general, these SULTs most effectively metabolize molecules containing a steroid or sterol nucleus (e.g., dehydroepiandrosterone (DHEA), pregnenolone, cholesterol, and bile acids) [26]. The SULT2A subfamily members, including human SULT2A1, are most highly expressed in liver, intestine, and adrenal cortex [27–30], and the prototypical SULT2A substrate is DHEA [27] (Figure 1). SULT2A-catalyzed DHEA sulfoconjugation within the adrenal cortex provides high circulating levels of DHEA sulfate, which serves as a reservoir of a precursor molecule that can be converted into potent androgens and estrogens within various tissues (e.g., prostate) [31].

The SULT2B subfamily consists of two gene products, SULT2B1a and SULT2B1b, that are transcribed from the same gene locus through the utilization of different promoters and incorporation of different first exons [11, 32, 33]. SULT2B1b preferentially catalyzes the sulfonation of cholesterol (Figure 1), and SULT2B1b expression has been demonstrated in skin [11, 33–35], prostate [11, 34, 36], placenta [34, 36], lung [34, 37], intestine [11, 33, 36], endometrium [36, 38], breast [39], ovary [36], platelets [40], kidney [33], and muscle [11]. SULT2B1b protein has not been detected in liver [34]. SULT2B1b has been detected in both the cytosol and nuclei of human cells [34, 39, 41].

By comparison to SULT2B1b, SULT2B1a has minimal activity toward cholesterol but readily catalyzes the sulfonation of pregnenolone [42]. Pregnenolone sulfate is a neurosteroid that is synthesized in glial cells [43]. It is, therefore, noteworthy that SULT2B1a mRNA expression has been detected in brain [11] as well as in rat C6 glioma cells [44]. However, to date SULT2B1a protein has not been detected in any human tissues or cell lines [41].

In addition to the major SULT1 and SULT2 enzymes, SULT3A1, SULT3A2, SULT4A1, SULT5A1, and SULT6B1 have been described [45–48] but are not highly characterized. Of these, only SULT4A1 and SULT6B1 have been identified in humans [48–50].

### 3.2. Regulation of SULTs by PPAR

#### 3.2.1. Regulation of Mouse Liver SULT Expression by PPAR*α* Agonists

A comprehensive survey of SULT regulation by PPAR*α*-activating treatments has been performed by the Klaassen research group, which evaluated the sex-dependent regulation of hepatic SULT expression following in vivo treatment of mice with a panel of prototypical nuclear receptor activators, including three PPAR*α* agonists [51]. Hepatic transcript levels were evaluated for murine SULTs 1a1, 1b1, 1c1, 1c2, 1d1, 1e1, 2a1/2a2, 2b1, and the lesser characterized SULT family members 3a1, 4a1, and 5a1 [51]. In addition, treatment effects on the mRNA expression of both forms of PAPS synthase (PAPSs1 and PAPSs2), the enzymes responsible for synthesizing the PAPS cofactor for sulfate conjugation, were examined [51]. Male and female 8 week old C57BL/6 mice were treated for 4 days with either corn oil vehicle or one of the prototypical PPAR*α* ligands, clofibrate (500 mg/kg IP), ciprofibrate (40 mg/kg IP), or diethylhexylphthalate (1000 mg/kg IP), and euthanized for hepatic SULT mRNA content analysis [51]. Overall, the effects of PPAR*α* activation on murine hepatic SULT expression were not striking [51]. SULT expression in male mouse liver was not appreciably perturbed in response to in vivo treatment with PPAR*α* agonists [51]. In the rat, SULT1E1, which is more abundantly expressed in male relative to female liver, was previously reported to decrease substantially following in vivo treatment with any of the PPAR*α* agonists, WY-14,643, gemfibrozil or di-*n*-butylphthalate [52]. In Klaassen's study, the mRNA levels of several SULTs were suppressed in female mouse liver following PPAR*α* agonist treatment, including SULTs 1c1, 1c2, 1e1, 2a1/2a2, 3a1, and 5a1 [51]. Treatment with clofibrate increased PAPSs2 mRNA content in male mouse liver [51]. However, such induction was not produced by ciprofibrate or diethylhexylphthalate treatment, suggesting that regulation of murine PAPSs2 expression is not an effect common to PPAR*α* agonists [51]. These studies demonstrate that PPAR*α* activation produces gene- and sex-dependent effects on the hepatic expression of SULT enzymes in the mouse.

#### 3.2.2. Transactivation of Human Hepatic SULT2A1 Transcription by PPAR*α*

The above-described data suggest that PPAR*α* does not function as a positive regulator of murine hepatic SULT expression. However, our laboratory has demonstrated that human hepatic SULT2A1 expression is increased by PPAR*α* activation, and that this effect is conferred through a functional PPAR*α*-response element (PPRE) located in the distal 5′-flanking region of the SULT2A1 gene [53]. The treatment of primary cultured human hepatocytes with ciprofibrate induced SULT2A1 mRNA, protein and enzyme activity levels by ~2-fold [53]. This finding was in marked contrast to the rat counterpart of SULT2A1, which was not inducible in primary cultured rat hepatocytes following treatment with a PPAR*α* agonist [53]. Analysis of a series of SULT2A1 5′-flanking region-luciferase reporter constructs in HepG2 cells revealed the presence of a functional direct repeat with one intervening nucleotide (DR-1) located at nucleotides −5949 to −5929 relative to the transcription start site [53] (Figure 2). Further site-directed mutagenesis, EMSA and chromatin immunoprecipitation analyses confirmed the functionality of this PPRE in the human SULT2A1 gene [53]. These investigations reveal that SULT2A1 represents a target for lipid signaling in human hepatocytes and suggest that rodent models do not capture the significance of PPAR*α* as a modulator of SULT2A expression.

#### 3.2.3. Regulation of SULT2B1b in Human Keratinocytes by PPAR Agonists

**Figure 3:**
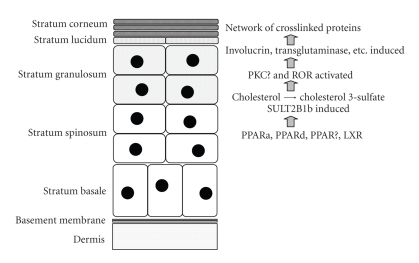
*Representation of keratinocyte differentiation and role of SULT2B1b-mediated cholesterol sulfonation*. During formation of the epidermis, replicating basal keratinocytes give rise to progeny that move progressively upwards and pass through several histologically distinct strata. During this process, SULT2B1b expression becomes activated at approximately the level of the stratum granulosum. SULT2B1b-catalyzed cholesterol sulfonation produces cholesterol 3-sulfate that, in addition to its role as a lipid component of the outer barrier, functions as a signaling molecule that activates PKC*η* and possibly ROR*α*. These signaling events result in the induction of proteins that are involved in formation of the barrier. PPAR*α*, PPAR*δ*, PPAR*γ*, and LXR have all been reported to positively regulate SULT2B1b expression in cultured keratinocytes.

Maintenance of the skin requires a well orchestrated program of keratinocyte proliferation and differentiation during which the cells pass through several phenotypic phases identifiable as distinct layers (Figure 3). As keratinocytes progress through this differentiation program, a major change that occurs is the production of progressively larger amounts of lipid. Cholesterol 3-sulfate has been detected at a level of 2.4% (weight percent of total lipids) in a combined preparation of basal/spinous cells, and 5.5% in the granular layer [54]. The amount of cholesterol 3-sulfate is then reduced in the stratum corneum, due to the conversion of cholesterol 3-sulfate to free cholesterol through the action of steroid sulfatase [54]. Cholesterol 3-sulfate production in the lower levels of the epidermis and its hydrolysis in the stratum corneum has been termed the epidermal cholesterol cycle [55]. 

In 1984, an enzyme capable of sulfonating cholesterol was reported to be present in mouse epidermis [56], and several subsequent pieces of evidence have supported the concept that “cholesterol sulfotransferase” expression and cholesterol 3-sulfate production are events that are closely linked to keratinocyte differentiation. For example, the specific activity of cholesterol sulfotransferase, but not of steroid sulfatase, paralleled both the accumulation of cholesterol 3-sulfate and the formation of the multilayered structure of the epidermis during mouse development [57, 58]. Cholesterol sulfotransferase activity was also induced during the culture of epidermal tissues isolated from 13.5-days, postcoitus mouse embryos with a time course that paralleled in vitro stratification [58]. In monolayer cultures of normal human keratinocytes, confluence-mediated differentiation was accompanied by increased cholesterol sulfotransferase activity and accumulation of cholesterol 3-sulfate, with parallel increases in transglutaminase-1 [59]. Calcium-mediated differentiation of normal human keratinocytes was accompanied by the induction of two distinct sulfotransferase activities, cholesterol sulfotransferase, and minoxidil sulfotransferase [60]. Exposure of fetal rat skin explants to air caused the accelerated expression of cholesterol sulfotransferase along with several other markers of keratinocyte differentiation, including filaggrin, loricrin and involucrin [61]. A single topical administration of a tumor promoter (e.g., phorbol ester) to mouse skin caused the induction of cholesterol sulfotransferase, the accumulation of cholesterol 3-sulfate, and the induction of transglutaminase-1 [62, 63]. Cholesterol sulfotransferase activity was also elevated during epidermal wound healing [62]. 

SULT2B1b preferentially sulfonates cholesterol and is the chief SULT2B1 enzyme that is expressed in human keratinocytes [64]. Higashi et al. [35] reported that SULT2B1b, but not SULT2B1a or SULT2A1, was present in normal human epidermal tissue, as well as in cultures of normal human keratinocytes following calcium-induced differentiation. By immunohistochemistry, SULT2B1b was largely localized to the granular layer of normal skin where cholesterol sulfate content is highest [35]. Altogether, the combination of substrate specificity and expression properties establish SULT2B1b as the “cholesterol sulfotransferase” of differentiating keratinocytes [35].

In addition to its role as structural component, cholesterol 3-sulfate is an important signaling molecule that plays an essential role in keratinocyte differentiation (Figure 3). For example, cholesterol 3-sulfate has been proposed to be a physiological activator of the novel protein kinase C (PKC) isoform, PKC*η*, which is localized to the granular layer where cholesterol sulfate concentrations are the highest [65–67]. Activation of PKC*η* signals the continuance of the keratinocyte differentiation program, resulting in the expression of late differentiation markers, such as transglutaminase1 [65, 66, 68, 69]. In addition, it has been demonstrated that cholesterol sulfate is a preferred ligand for the retinoid-related orphan nuclear receptor-*α* (ROR-*α*). This nuclear receptor, particularly the ROR*α*4 splice variant, is robustly expressed in the epidermis, where it is present in suprabasal differentiating keratinocytes but not in proliferating basal keratinocytes [70]. Until recently, ROR*α* was considered to be a true orphan receptor, since no firm ligand activator for ROR*α* had been identified. However, recent X-ray crystallography data revealed the presence of either cholesterol or cholesterol sulfate within the ROR*α* ligand binding pocket domain [70]. Furthermore, pharmacological manipulation of cellular cholesterol levels altered ROR*α* transcriptional activity in human osteosarcoma cells [70]. These studies led to the recognition that ROR*α* may represent a novel regulatory pathway in the control of cholesterol homeostasis [71]. Compared to cholesterol, cholesterol sulfate demonstrated a greater affinity for the ROR*α* ligand binding domain and more profound effects on ROR*α*-mediated transcriptional activation, suggesting a major role for cholesterol sulfate as a physiologic activator of ROR*α* in tissues such as the epidermis where both cholesterol sulfate and ROR*α* are abundantly coexpressed [71, 72]. 

In light of the functional role of SULT2B1b in keratinocyte differentiation, corneoctye desquamation and dermal lipid homeostasis, it is reasonable to expect that SULT2B1b should be a target for regulation by lipid-sensing nuclear receptors in the skin. The effects, on SULT2B1b expression, of treating cultured human keratinocytes with chemical activators of PPAR*α*, PPAR*δ*, PPAR*γ*, and of the liver X receptor (LXR*α* and LXR*β*) have been examined [73]. Measurable levels of PPAR and LXR transcripts were detected in cultured human keratinocytes, and the levels of PPAR*γ*, LXR*α*, and LXR*β* increased following calcium-induced differentiation [73]. By contrast, PPAR*α* and PPAR*δ* mRNA levels did not change demonstrably with keratinocyte differentiation [73]. Treatment with PPAR*α*, PPAR*δ*, PPAR*γ*, and LXR agonists significantly induced SULT2B1b mRNA and enzyme activity in cultured human keratinocytes [73]. The PPAR*α* agonist clofibric acid increased SULT2B1b mRNA content by ~39.9% [73]. Activators of PPAR*δ* (GW501516) and PPAR*γ* (ciglitazone) produced even greater increases (~9.8-fold and ~25.1-fold, resp.) in SULT2B1b mRNA content, and these increases were further augmented by calcium-induced keratinocyte differentiation [73]. As modulators of SULT2B1b expression in differentiating keratinocytes, these results underscore the central role of PPAR transcription factors as integrators of skin physiology and barrier function (Figure 3).

## 4. UDP-Glucuronosyltransferases

### 4.1. The Role of UGTs in Metabolism

The UGTs catalyze the transfer of glucuronic acid from a high-energy cofactor, UDP-glucuronic acid, to a xenobiotic or endogenous substrate containing an available nucleophilic center such as a hydroxyl, carboxyl, amino, or thiol group [74–77] (Figure 1). The UGTs are membrane-bound enzymes localized on the luminal surface of the endoplasmic reticulum [74]. Relative to the parent substrate, the end-products of glucuronidation are typically more polar and better suited for excretion and elimination through the urine or bile [74]. Endogenous UGT substrates include bilirubin, neutral steroids, bile acids, fatty acids, and retinoids [74, 78, 79]. Xenobiotic UGT substrates range from environmental toxicants such as benzo[a]pyrene to common pharmaceuticals such as acetaminophen, nonsteroidal anti-inflammatory agents, fibrates, thiazolidinedione-class insulin sensitizers, and opioids [75, 78–84]. Individual UGT enzymes display distinctive patterns of substrate specificity and inducible regulation, but as with the SULTs, some UGTs display overlapping substrate specificities [74, 85]. Different UGTs are expressed in a species- and tissue-specific manner [86, 87]. Overall, the broad metabolic range of the UGTs distinguishes this class of conjugating enzyme as a major detoxicating system in rodents and humans.

Though over 50 individual UGT enzymes have been described [86, 88], comparisons of cDNA and amino acid sequences have revealed two major UGT gene families, UGT1 and UGT2 [75, 86, 88, 89]. The UGT1A multigenic locus is unusual in that it is comprised of a tandem series of thirteen promoter regions on human chromosome 2 [75, 86, 90]. Each of nine functional UGT1A proteins is produced as a result of transcription initiation at a particular promoter, which results in the transcription and splicing of a unique exon 1 sequence to a cassette of common exons (exons 2–5) that share the same 3′ end [75, 86, 88, 90, 91]. As an additional layer of complexity, recent evidence indicates that alternative splicing events generate UGT1A isoforms with different exon 5 sequences. For each of these UGT1A proteins, isoform 1 (containing exon 5a) is the classical catalytically active enzyme, while isoform 2 (containing exon 5b) is a smaller protein that lacks UGT activity but can inhibit the activity of the corresponding isoform 1 [92, 93]. By contrast to the UGT1A locus, UGT2 enzymes are products of individual genes [75].

Like sulfonation, glucuronidation plays a physiological role in the modulation of biologically active endogenous hormones and metabolic intermediates. For example, the activity of UGT1A1 conjugation in the tight control of bilirubin metabolism has established the importance of UGT1A1 genetic polymorphisms in the pathogenesis of toxic hyperbilirubinemias such as the Crigler-Najjar and Gilbert's syndromes in humans [75, 89, 94]. Thyroxine is metabolized by O-glucuronidation in addition to deiodination and sulfonation [95]. Studies using recombinant UGTs and human liver microsomes revealed that human hepatic UGT1A1 and UGT1A3 are the UGT principals most catalytically active toward thyroxine [95]. Bile acids represent the end-products of hepatic cholesterol metabolism and in the absence of sufficient detoxication metabolism, particularly in the face of cholestasis, the detergent properties of bile acids produce significant hepatotoxicity [96]. Human UGT2B4 [97], UGT2B7 [96, 98], UGT1A4 [96, 98], and UGT1A3 [98, 99] are all bile acid-metabolizing enzymes. The formation of chenodeoxycholic acid (CDCA) glucuronide by UGT1A3 has been shown to decrease farnesoid X receptor (FXR) activation by CDCA, the prototypical FXR ligand [99], suggesting that UGT1A3 induction in human liver would be expected to have down-stream consequences for an array of gene expression networks that are transcriptionally regulated by FXR.

### 4.2. Regulation of UGTs by PPAR

#### 4.2.1. Studies of the Effects of Clofibrate and Other Perixosome Proliferation-Inducing Agents on UGT Activities

It has been more than 40 years since the discovery that clofibrate treatment causes peroxisome proliferation in rat liver [100, 101]. After clofibrate was subsequently shown to cause hepatic induction of a unique cytochrome P450 with a reduced carbon monoxide-bound absorbance peak at 452 nm and catalytic activity toward lauric acid 12-hydroxylation, clofibrate became accepted as the prototype of a novel class of microsomal enzyme inducers [102]. Clofibrate, therefore, was used in a number of studies designed to evaluate the effects of microsomal enzyme inducer treatments on glucuronidation activities. The most commonly reported finding in these studies was that treatment with clofibrate or a structurally related compound increased hepatic glucuronidation activity toward bilirubin in rats [103–110], mice [111], and primary cultured rat hepatocytes [112], with the clofibrate-mediated increases generally in the range of two-fold over control. In some of these studies, use of multiple compounds provided evidence for correspondence between induction of rat hepatic bilirubin conjugation activity and induction of lauric acid 12-hydroxylation [104, 105], implying probable identity of the induction mechanism. In addition, human subjects with Gilbert's syndrome treated with clofibrate had decreased serum total bilirubin concentrations [113], and hepatic microsomes prepared from humans who had received clofibrate contained elevated bilirubin UGT activity [114].

Clofibrate treatment of rats has also been reported to increase hepatic UGT activity toward 4′-hydroxy-N,N-dimethyl-4-aminoazobenzene [106], thyroxine and reverse triiodothyronine [109, 115], retinoic acid [116], and the antithrombotic drug LF 4.0212 [117]. In agreement with these findings, clofibric acid treatment also increased thyroxine UGT activity in primary cultured rat hepatocytes [112]. However, a species difference in UGT induction was suggested by the finding that clofibrate treatment of male OF-1 mice displayed no increase in hepatic microsomal thyroxine UGT activity [111]. Likewise, clofibric acid treatment of primary cultured mouse hepatocytes (OF-1 strain) did not increase bilirubin or thyroxine UGT activity [112]. In a porcine model, in vivo treatment with clofibrate induced hepatic glucuronidation of thyroid hormones sufficiently to reduce circulating plasma 3,3′,5-triiodothyronine and thyroxine concentrations [118].

Clofibrate treatment has been consistently reported not to increase UGT activity toward *p*-nitrophenol in rat liver [106, 109, 110], mouse liver [111], primary cultured rat hepatocytes [112, 119], or primary cultured mouse hepatocytes [112]. Clofibrate treatment of rats or mice (OF-1 strain) has also been shown to have little effect on hepatic microsomal UGT activities toward triiodothyronine and androsterone [109, 111].

In another study, treatment of rats with a single dose of the nonfibrate peroxisome proliferator, the fully-fluorinated ten-carbon fatty acid perfluorodecanoic acid, induced hepatic bilirubin UGT activity two-fold, and this induced a state persisted for 3 weeks [120]. This single-dose treatment also decreased hepatic UGT activities toward 1-naphthol, morphine, and testosterone, with maximal reductions occurring 12 days after treatment and recovery to control activities occurring at 3 weeks [120].

These early findings, in which peroxisome proliferator treatments produced differential effects on various UGT activities, provided a clear indication of the multiplicity of the UGT superfamily. As seen for the cytochromes P450, only certain UGT activities displayed peroxisome proliferator inducibility, predicting the later demonstration that particular UGT genes would be targets of PPAR*α*-mediated transactivation.

#### 4.2.2. UGT1A Regulation by PPARs

UGT1A1 is the major catalyst of bilirubin glucuronidation [121] (Figure 1). Therefore, based on the above-described observation that clofibrate treatment consistently caused induction of hepatic bilrubin UGT activity, UGT1A1 is expected to be a PPAR*α* target gene, possibly along with other UGTs of the “bilirubin-like” portion of the UGT1A subfamily (i.e., UGTs 1A2-1A5). 

In a study using antipeptide antibodies to examine the effects of drug treatments on rat hepatic microsomal UGT levels, clofibrate treatment was found to increase the immunoreactive protein levels of UGT1A1 and UGT1A5 (termed UGT1B1 and UGT1B5 in that study) along with bilirubin UGT activity [110]. In a more recent study, four-day treatments of male rats with PPAR*α* activators produced modest increases in hepatic UGT1A1 and UGT1A3 mRNA levels [122]. In addition, clofibric acid treatment of primary cultured rat hepatocytes has been reported to increase the amount of UGT1A1 protein by western blot analysis [119] and the amount of UGT1A1 mRNA by microarray analysis [123]. Therefore, as predicted by the bilirubin glucuronidation activity data, UGT1A1 is a target of PPAR*α* activation. Clofibrate treatment has also been reported to decrease the amount of rat hepatic microsomal UGT1A6 protein (termed UGT1A1 in that study) [110]. This finding is also in agreement with the above-mentioned earlier finding that clofibrate treatment did not induce *p*-nitrophenol glucuronidation activity in rodent liver or cultured hepatocytes, since UGT1A6 is a major catalyst of that activity [124] (Figure 1).

In a recent study by Senekeo-Effenberger et al. [125], Wy-14,643 treatment of primary cultured human hepatocytes increased the levels of UGT1A1, UGT1A3, UGT1A4, and UGT1A6, but not UGT1A9, mRNAs. In the same study, experiments conducted with transgenic mice engineered to express the complete human UGT1 gene locus demonstrated that oral Wy-14,643 treatment resulted in prominent induction of human UGT1A1 and UGT1A6, and observable induction of UGT1A4, immunoreactive protein content in liver microsomes. At the mRNA level in liver, very strong (>100-fold) induction of UGT1A1 and UGT1A3, significant (~3- to 4-fold) induction of UGT1A4 and UGT1A6, and no induction of UGT1A9 were seen, in general agreement with the above-described effects in the human hepatocyte cultures [125]. In small intestine, Wy-14,643 treatment produced induction of UGT1A1 and UGT1A4, but not UGT1A6, while in kidney only UGT1A6 was induced [125]. Additional experiments confirmed the presence of functional PPREs in the 5′-flanking regions of UGT1A1, UGT1A3 and UGT1A6 [125] (Figure 2).

In another study, treatment of primary cultured human hepatocytes with activators of PPAR*α* increased the expression of UGT1A3 and UGT1A3-catalyzed glucuronidation of chenodeoxycholic acid (CDCA) and demonstrably tempered the transactivation of FXR by CDCA [99]. Promoter analysis of the human UGT1A3 gene revealed coregulation by two lipid-sensing transcription factors, LXR [126] and PPAR*α* [125].

UGT1A4 was also identified as a significantly upregulated gene in clofibric acid-treated primary cultured human hepatocytes by Affymetrix microarray analysis [123]. In this same study, clofibric acid treatment of primary cultured mouse hepatocytes failed to cause significant alteration of any UGT transcripts [123], consistent with the above-described apparent lack of sensitivity of mouse to PPAR*α*-mediated regulation of UGT activities.

By comparison to the results described by Senekeo-Effenberger et al. [125], Barbier et al. [78] previously reported that mouse and human UGT1A9 are transcriptionally activated by PPAR*α* and PPAR*γ*, and that human UGT1A9 contains a functional PPRE located at nucleotides −719 to −706 (Figure 2). UGT1A9 mRNA induction was observed in primary cultured human hepatocytes treated with fenofibric acid, HepG2 cells treated with Wy-14,643, 3T3-L1 adipocytes treated with rosiglitazone, primary human macrophages treated with Wy-14,643, or differentiated THP-1 macrophages treated with Wy-14,643 or rosiglitazone [78]. As expected, the inducing effect of fenofibric acid on mouse hepatic UGT1A9 was ablated in PPAR*α*-null mice [78].

#### 4.2.3. UGT2B Regulation by PPAR*α*

In human hepatocytes, treatment with a PPAR*α* agonist, fenofibric acid or Wy-14643, increased the expression of UGT2B4 mRNA and stimulated the glucuronidation of hyodeoxycholic acid, a model substrate for UGT2B4 [97] (Figure 1). Transient transfection and EMSA studies revealed a functional DR-1 PPRE at nucleotides −1199 to −1175 nt relative to the UGT2B4 transcription start site and solidified UGT2B4 as a transcriptional target of PPAR*α* [97] (Figure 2).

## 5. Regulation of SULTs and UGTs by Other Nuclear Receptors

In addition to regulation by PPARs, the SULTs and UGTs receive transcriptional input from multiple other nuclear receptors. Here, we do not attempt to be comprehensive but present several findings related to the regulation of hepatic SULT2A expression as an example. Our previous investigations suggested roles for both the glucocorticoid receptor (GR) and the pregnane X receptor (PXR) in the mediation of glucocorticoid-inducible rat hepatic SULT2A expression [127]. GR-activating concentrations of glucocorticoid transactivated SULT2A transcription indirectly, through intermediary steps involving GR-inducible liver-enriched CCAAT enhancer binding protein [128], while pharmacological concentrations of dexamethasone induced rat hepatic SULT2A expression via a PXR-mediated mechanism [127]. Rodent and human SULT2A are differentially regulated by the xenobiotic-sensing receptor, PXR. In mice and rats, hepatic SULT2A transcription is activated by PXR through the direct binding of PXR to the 5′-flanking regions of SULT2A genes [127, 129]. However, unlike rodent hepatic SULT2As, treatment of human liver cells with rifampicin, the prototypical ligand activator of human PXR [130], produces a biphasic effect on SULT2A1 expression [131]. Treatment with PXR-activating concentrations of rifampicin causes PXR-dependent suppression of SULT2A1 expression, whereas treatment with higher rifampicin concentrations induces SULT2A1 expression through a PXR-independent mechanism [131]. In addition, the nuclear receptor hepatocyte nuclear factor 4*α* (HNF4*α*) is a positive regulator of SULT2A1 expression, and both the suppressive and activating effects of rifampicin appear to be transduced through interactions with HNF4*α* [131].

The constitutive androstane receptor (CAR) also partners with RXR and transactivates murine hepatic SULT2A, and possibly human SULT2A1 [132]. The vitamin D receptor (VDR, NR1I1) is activated not only by 1*α*,25-dihydroxyvitamin D_3_ but also by bile acids [133], and emerging evidence suggests that the VDR regulates murine hepatic SULT2A transcription, and can also drive the transcription of SULT2A1 in vitro [134]. The role of SULT2A as an integrator of endogenous lipid metabolism is just emerging. Oxysterol intermediates of cholesterol metabolism are physiological ligands for LXR, an RXR heterodimer transcription factor that regulates a number of genes involved in the maintenance of lipid homeostasis [135]. LXR-inducible murine hepatic SULT2A gene transcription has been described and has been shown to confer a protective effect against bile acid toxicity during cholestasis [136]. Certain sulfonated auto-oxidized sterols, such as 5*α*,6*α* epoxycholesterol-3-sulfate and 7-ketocholesterol-3-sulfate, have been shown to interact with the LXR*α* ligand-binding domain and inhibit LXR*α*-mediated transactivation in vitro [137]. A suppressive role for FXR in the regulation of hepatic SULT2A expression has also been identified, as SULT2A expression was increased 8 fold in FXR-null mice as compared to wild-type mice, and CDCA feeding decreased SULT2A expression in wild-type mice [138]. Also, SULT2A1 expression was reduced following treatment of HepG2 cells with FXR agonists [138].

## 6. Conclusions

Emerging evidence supports an under-appreciated physiological role for members of the SULT and UGT gene families to serve as modulators of biologically active lipids and to undergo transactivation by lipid-sensing transcription factors such as the PPARs. Particularly in keratinocytes which rely on lipid signaling for the progression of programmed cellular differentiation, the inducible expression of cholesterol sulfotransferase (SULT2B1b) by PPAR activators has been demonstrated. Studies in primary cultured human but not rat hepatocytes clearly demonstrate that PPAR*α*-inducible human hepatic SULT2A1 expression occurs through a distal PPRE in the 5′-flanking region of the SULT2A1 gene. Several human hepatic UGTs also demonstrate inducible transcription in response to PPAR*α* activation, and transgenic mice expressing the human UGT1 gene locus display transcriptional regulation of human UGT1A transgenes in liver and intestine by PPAR*α*. In view of the coordinated integration between phase II metabolism and PPAR lipid signaling networks, future investigations will likely focus on the disturbances in hepatic and gastrointestinal lipid homeostasis that significantly alter SULT and UGT expression sufficiently to disrupt the down-stream metabolism of environmental xenobiotics, pharmaceuticals, or biologically active intermediates of metabolism.
